# Oral health in HPV-positive and HPV-negative patients with oropharyngeal squamous cell carcinoma

**DOI:** 10.3389/fonc.2023.1083067

**Published:** 2023-01-26

**Authors:** Philipp Kanzow, Katharina Mielke, Valentina Hrasky, Susanne Wiegand, Henning Schliephake, Dirk Beutner, Annette Wiegand

**Affiliations:** ^1^ Department of Preventive Dentistry, Periodontology and Cariology, University Medical Center Göttingen, Göttingen, Germany; ^2^ Department of Otolaryngology, Head and Neck Surgery, University of Leipzig, Leipzig, Germany; ^3^ Department of Oral and Maxillofacial Surgery, University Medical Center Göttingen, Göttingen, Germany; ^4^ Department of Otorhinolaryngology, Head and Neck Surgery, University Medical Center Göttingen, Göttingen, Germany

**Keywords:** head and neck cancer, head and neck squamous cell carcinomas, HPV, oral health, oropharynx cancer, survival

## Abstract

This study compared oral health in oropharyngeal squamous cell carcinoma (OPSCC) patients with positive or negative human papillomavirus (HPV) status and analysed whether oral health was associated with survival. Patients referred for dental assessment prior to radio(chemo)therapy between 2009 and 2019 were included. Patient-related risk factors for OPSCC (alcohol, tobacco, HPV status), age, sex, treatment (primary treatment, intent), performance status, tumor/node/metastasis (TNM) staging, and oral health parameters (DMFT, periodontal status, teeth with/without root canal treatment and with/without periodontitis apicalis) were compared between HPV-negative and HPV-positive patients. Survival was assessed using Kaplan-Meier statistics. The effect of patient-related risk factors and oral health parameters was analysed by cox regression analyses (α=5%). A total of 119 patients (n=50 HPV-negative, n=69 HPV-positive) was included. HPV-positive patients showed more present teeth, a higher number of filled teeth, were less often edentulous and presented a lower DMFT compared to HPV-negative patients (p_adj._≤0.003). Among dentulous patients, HPV-positive patients showed more present teeth and fewer teeth with periodontitis apicalis lacking a root canal treatment (p_adj._≤0.036). Survival probability differed between groups (p=0.006) and trended towards being associated with HPV status, tobacco exposure, performance status, T stage, N stage, and the number of missing or filled teeth as well as the number of root canal treated teeth with periodontitis apicalis and the number of teeth with periodontitis apicalis lacking a root canal treatment (p≤0.077). However, only tobacco exposure, performance status, and the number of teeth with periodontitis apicalis lacking a root canal treatment in dentulous patients remained significant in the multivariate analyses (p≤0.047). HPV-negative patients with OPSCC showed a poorer oral health compared to HPV-positive patients, but survival was not associated with oral health.

## 1 Introduction

Oral health is often compromised in patients with head and neck squamous cell carcinoma (HNSCC) (1–4). Several previous studies found a positive association between poor oral health or poor oral hygiene and HNSCC (5–7). This association can be attributed to different mechanisms: Oral diseases, especially periodontitis, and HNSCC have shared risk factors, like tobacco exposure and alcohol consumption. It is furthermore discussed that oral hygiene measures and good oral health are surrogate parameters for general health or a healthier lifestyle, the latter preventing the development of cancer (5, 8, 9). More recent studies adjusted for confounding factors also indicated a direct effect of periodontitis on HNSCC: For instance, increased levels of free radicals, cytokines or chemokines that are generated during the inflammatory process by inflammatory cells and/or periodontal pathogens in the oral microbiome might induce DNA damage or mutation (10).

Besides classic risk factors, infection with high-risk genotypes of the human papillomavirus (HPV) was identified as risk factor for HNSCC, especially for oropharyngeal squamous cell carcinoma (OPSCC). HPV-positive OPSCC is a distinct tumor entity with different clinical, histopathological, and molecular characteristics (11). Patients with HPV-positive OPSCC were younger, have lower alcohol and tobacco exposure, and a better prognosis compared to HPV-negative patients (12). It is thus possible that the association between oral health and OPSCC is different in both subsets, potentially having consequences for the dental treatment prior, during, and after radio(chemo)therapy.

With regard to previous studies, Mazul et al. (13) found a similar pattern of association between poor oral health and both HPV-positive and HPV-negative OPSCC; however, oral health was assessed by self-reported oral health variables rather than by clinical examination. Conflicting results were found when alveolar bone loss as a marker for periodontal disease was assessed in dental panoramic radiographs: Patel et al. (14) found that horizontal bone loss was less severe in HPV-positive compared to HPV-negative OPSCC, while Tezal et al. (15) found higher bone loss in HPV-positive patients with HNSCC.

Due to the limited number of studies and the inconsistent results, this study aimed to compare oral health in patients with HPV-negative and HPV-positive OPSCC. Furthermore, as survival of OPSCC patients depends on HPV status (16–18), this study also aimed to analyse whether oral health in HPV-positive and HPV-negative patients as well as in all patients is associated with survival.

## 2 Materials and methods

This study was approved by the Ethics committee of the University Medical Center Göttingen (17/10/19). In this retrospective single-centre study, dental records of OPSCC patients referred to the Department of Preventive Dentistry, Periodontology and Cariology of the University Medical Center Göttingen for dental assessment prior to planned radio(chemo)therapy between 2009 and 2019 were included. Dental assessments were performed to determine dental health prior to cancer treatment. If required, dental treatment (i.e. extractions in most cases) was scheduled/recommended.

Potentially eligible patients from the Department of Oral and Maxillofacial Surgery and the Department of Otorhinolaryngology, Head and Neck Surgery were automatically screened by different ICD-10 codes (C00-C14, D37.0, D37.9). Subsequently, both electronic and paper-based health records were manually screened in full, and patients not meeting the inclusion criteria were excluded. The inclusion criteria were as follows:

(1) Patient with OPSCC.

(2) Tumor treatment within the Department of Oral and Maxillofacial Surgery or the Department of Otorhinolaryngology, Head and Neck Surgery of the University Medical Center Göttingen.

(3) Dental assessment at the Department of Preventive Dentistry, Periodontology and Cariology of the University Medical Center Göttingen prior to planned radio(chemo)therapy between 2009 and 2019.

(4) Known HPV status.

The following patient-related data were obtained from patient files: age at the time of diagnosis, sex, tobacco exposure (never smoker vs. former/current smoker), alcohol consumption (no vs. previously/currently), HPV status (HPV-negative vs. HPV-positive as detected by immunohistochemistry), primary treatment (surgery +/- adjuvant radiotherapy vs. radiotherapy/radiochemotherapy), treatment intent (curative vs. palliative), performance status, and tumor/node/metastasis (TNM) staging.

To assess dental health, the number of decayed (D), missing (M), and filled (F) teeth were extracted from dental records and validated using the intra-oral or panoramic radiographs. Data on periodontal health (i.e. averaged radiographic bone loss, presence of subgingival calculus, radiographic furcation involvement) were taken from intra-oral or panoramic radiographs (19). Furthermore, the number of teeth with and without root canal treatment and the number of teeth with or without periodontitis apicalis were taken from intra-oral or panoramic radiographs (20). For each patient, the averaged radiographic bone loss across all teeth was measured and calculated as published previously (21). Bone loss was expressed as absolute (mm) and relative (%) values. Also, bone loss as a function of age was calculated (%/age). Data extraction and evaluation of radiographs was performed by a single calibrated examiner (K.M.). In a subsample of radiographs (n=20), radiographic bone loss was also evaluated by a second examiner (P.K.) and reassessed by K.M. after several weeks to calculate both inter-rater and intra-rater reliability.

### 2.1 Statistical analysis

Patient-related risk factors for OPSCC, age, sex, primary treatment, treatment intent, TNM staging, and oral health parameters were compared between HPV-negative and HPV-positive patients using Fisher’s exact test (dichotomous variables) or Wilcoxon rang-sum test (ordinal or continuous variables). P-values were adjusted for multiple testing according to Benjamini & Hochberg (α=5%).

Survival was assessed using Kaplan-Meier statistics and compared between HPV-negative and HPV-positive patients by log-rank test. Survival time was calculated from the date of diagnosis until event (death) or last known visit to the hospital (censoring). For all patients and separately for HPV-negative and HPV-positive patients, the effect of patient-related risk factors, age, sex, and oral health parameters was first analysed by univariate cox regression analyses and likelihood ratio test. Subsequently, only variables with a p-value ≤0.1 within the univariate analyses (i.e. variables which trended towards being associated with survival) were entered in multivariate cox regression analyses (α=5%). Inter-rater reliability between both examiners was assessed using two-way, agreement, average intraclass correlation [ICC(A,2)], while two-way, agreement, single intraclass correlation [ICC(A,1)] was used to determine intra-rater reliability (22).

All analyses were performed using the software R (version 4.2.1; www.r-project.org) and the packages “irr” (version 0.84.1) for assessment of intraclass correlation as well as “survminer” (version 0.4.9) and “survival” (version 3.4-0) for the time-to-event analyses.

## 3 Results

**Figure 1:**
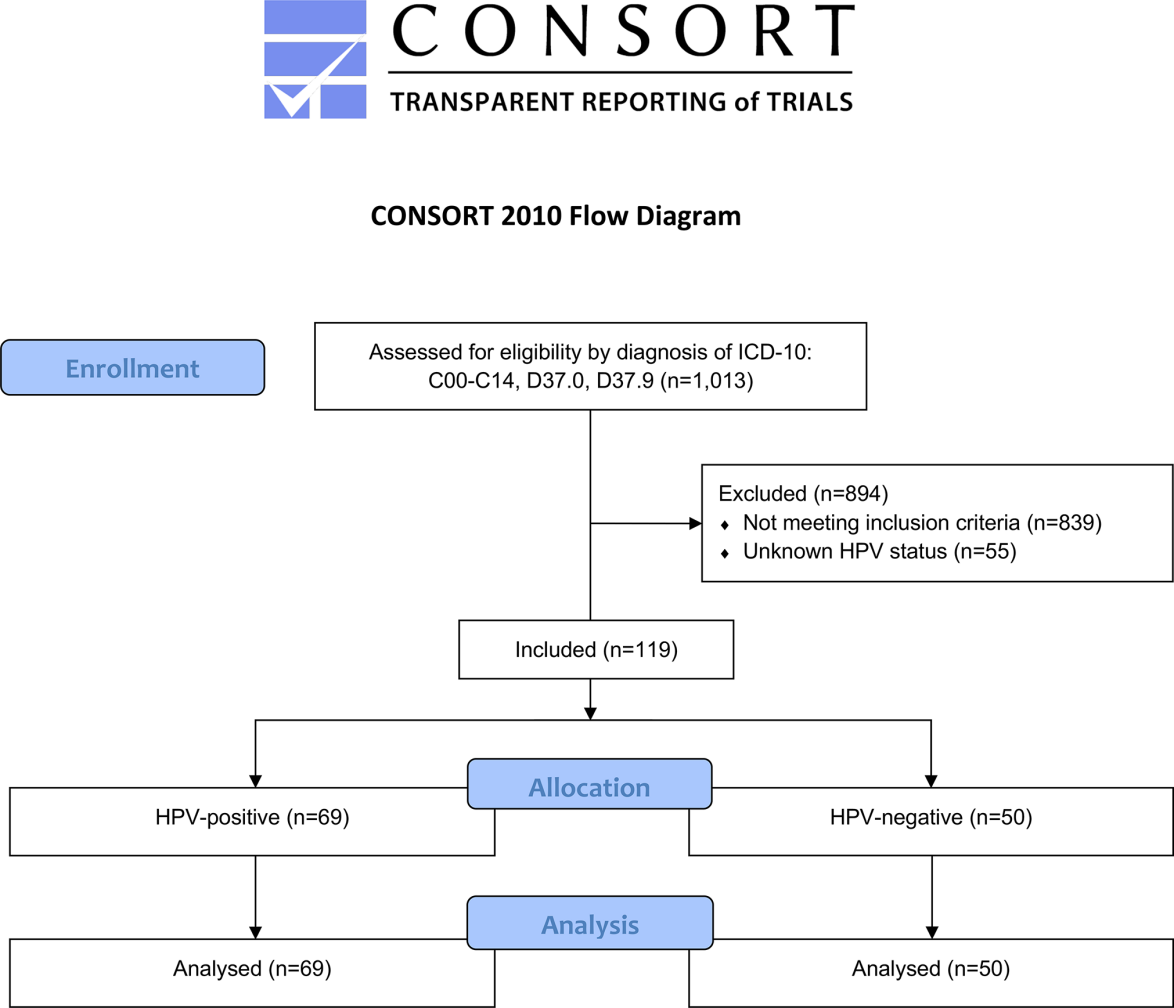
Modified CONSORT flow diagram of patient selection.

A total of 1,013 patients were screened for eligibility. Among these, 119 patients (n=50 HPV-negative, n=69 HPV-positive, mean age: 63.9±10.4 years) were included. The Consolidated Standards of Reporting Trials (CONSORT) flow diagram is shown in **Figure 1**.

The median follow-up time amounted to 2.6 years (min: 0.0, max: 9.6 years). HPV-negative patients were significantly more often former/current smokers (p_adj._<0.001), were less frequently treated with primary surgery (p_adj._=0.049), showed a lower performance status (p_adj._<0.001), and presented higher T stages (p_adj._=0.012). Alcohol consumption was also more often observed in HPV-negative compared to HPV-positive patients, but this difference was not significant (p_adj._=0.052).

With regard to oral health, HPV-positive patients showed significantly more present teeth (p_adj._<0.001), a higher number of filled teeth (p_adj._<0.001), were less often edentulous (p_adj._=0.003), and presented a lower DMFT (p_adj._<0.001) compared to HPV-negative patients (**Table 1**). Among dentulous patients (n=103), HPV-positive patients showed significantly more present teeth (p_adj._=0.036) and fewer teeth with periodontitis apicalis lacking a root canal treatment (p_adj._=0.012).

**Table 1:** Univariate comparison of patient-related risk factors for oropharyngeal squamous cell carcinoma, age, sex, primary treatment, treatment intent, tumor/node/metastasis staging, and oral health parameters between positive and negative human papillomavirus (HPV) status patients.

			Total(n=119)	HPV-negative(n=50)	HPV-positive(n=69)	p_adj._
Age at diagnosis, mean±SD			63.9 ± 10.4	65.2 ± 10.6	62.9 ± 10.3	0.323
Sex, n (%)	female		28 (23.5)	10 (20.0)	18 (26.1)	0.552
	male		91 (76.5)	40 (80.0)	51 (73.9)	
**Tobacco exposure, n (%)**	never smoker		56 (47.1)	13 (26.0)	43 (62.3)	<0.001 *
	former / current smoker		63 (52.9)	37 (74.0)	26 (37.7)	
Alcohol consumption, n (%)	never		85 (71.4)	30 (60.0)	55 (79.7)	0.052
	former / current		34 (28.6)	20 (40.0)	14 (20.3)	
**Primary treatment, n (%)**	surgery +/- adj. radiotherapy		100 (84.0)	37 (74.0)	63 (91.3)	0.049 *
	radiotherapy / radiochemotherapy		19 (16.0)	13 (26.0)	6 (8.7)	
Treatment intent, n (%)	curative		113 (95.0)	45 (90.0)	68 (98.6)	0.132
	palliative		6 (5.0)	5 (10.0)	1 (1.4)	
**Performance status, mean±SD *(n=109)***			88.8 (11.2)	84.4 (11.1)	92.3 (10.1)	<0.001 *
**T stage, n (%)**	T1		25 (21.0)	6 (12.0)	19 (27.9)	0.012 *
	T2		37 (31.1)	13 (26.0)	24 (35.3)	
	T3		34 (28.6)	17 (34.0)	17 (25.0)	
	T4		23 (19.3)	14 (28.0)	9 (13.2)	
N stage, n (%) *(n=118)*	N0		13 (11.0)	6 (12.0)	7 (10.3)	0.552
	N1		19 (16.1)	5 (10.0)	14 (20.6)	
	N2		80 (67.8)	37 (74.0)	43 (63.2)	
	N3		6 (5.1)	2 (4.0)	4 (5.9)	
M stage, n (%) *(n=111)*	M0		108 (97.3)	44 (97.8)	64 (97.0)	0.552
	M1		3 (2.7)	1 (2.2)	2 (3.0)	
**DMFT, mean±SD**	D		2.9 ± 4.1	3.9 ± 4.9	2.1 ± 3.2	0.275
	**M**		10.6 ± 9.5	14.5 ± 10.1	7.8 ± 8.0	<0.001 *
	**F**		7.7 ± 6.3	4.9 ± 5.6	9.8 ± 6.0	<0.001 *
	**DMFT sum**		21.2 ± 5.7	23.3 ± 5.9	19.7 ± 5.0	<0.001 *
**Number of teeth per patient, mean±SD**			17.4 ± 9.5	13.5 ± 10.1	20.2 ± 8.0	<0.001 *
**Edentulous patients, n (%)**			16 (13.4)	13 (26.0)	3 (4.3)	0.003 *
**Number of teeth in dentulous patients (n=103), mean±SD**			20.1 ± 7.1	18.2 ± 7.1	21.2 ± 6.9	0.036 *
Root canal treated teeth in dentulous patients, mean±SD *(n=99)*	all		1.3 ± 1.6	1.1 ± 1.5	1.4 ± 1.7	0.552
	without periodontitis apicalis		0.6 ± 1.3	0.6 ± 1.1	0.7 ± 1.4	0.993
	with periodontitis apicalis		0.5 ± 0.8	0.4 ± 0.8	0.5 ± 0.9	0.414
**Number of teeth with periodontitis apicalis but without root canal treatment, mean±SD *(n=99)***			0.7 ± 1.4	1.3 ± 1.8	0.4 ± 0.9	0.012 *
Averaged radiographic bone loss in dentulous patients, mean±SD *(n=99)*		mm	3.6 ± 1.8	3.9 ± 1.7	3.4 ± 1.8	0.126
		%	12.0 ± 10.5	13.9 ± 10.4	10.9 ± 10.5	0.088
		%/age	0.2 ± 0.2	0.3 ± 0.2	0.2 ± 0.2	0.058
Presence of subgingival calculus *(n=99)*		yes	36 (36.4)	15 (41.7)	21 (33.3)	0.552
		no	63 (63.6)	21 (58.3)	42 (66.7)	
Radiographic furcation involvement *(n=99)*		yes	45 (45.5)	19 (52.8)	26 (41.3)	0.410
		no	54 (54.5)	17 (47.2)	37 (58.7)	

For some variables, the number of patients is reduced due to missing data / radiographs. * p<0.05.

Inter-rater and intra-rater reliability of averaged radiographic bone loss measurements (%) amounted to 0.946 and 0.998, respectively. Thus, inter-rater and intra-rater reliability can be regarded as excellent (23).

**Figure 2:**
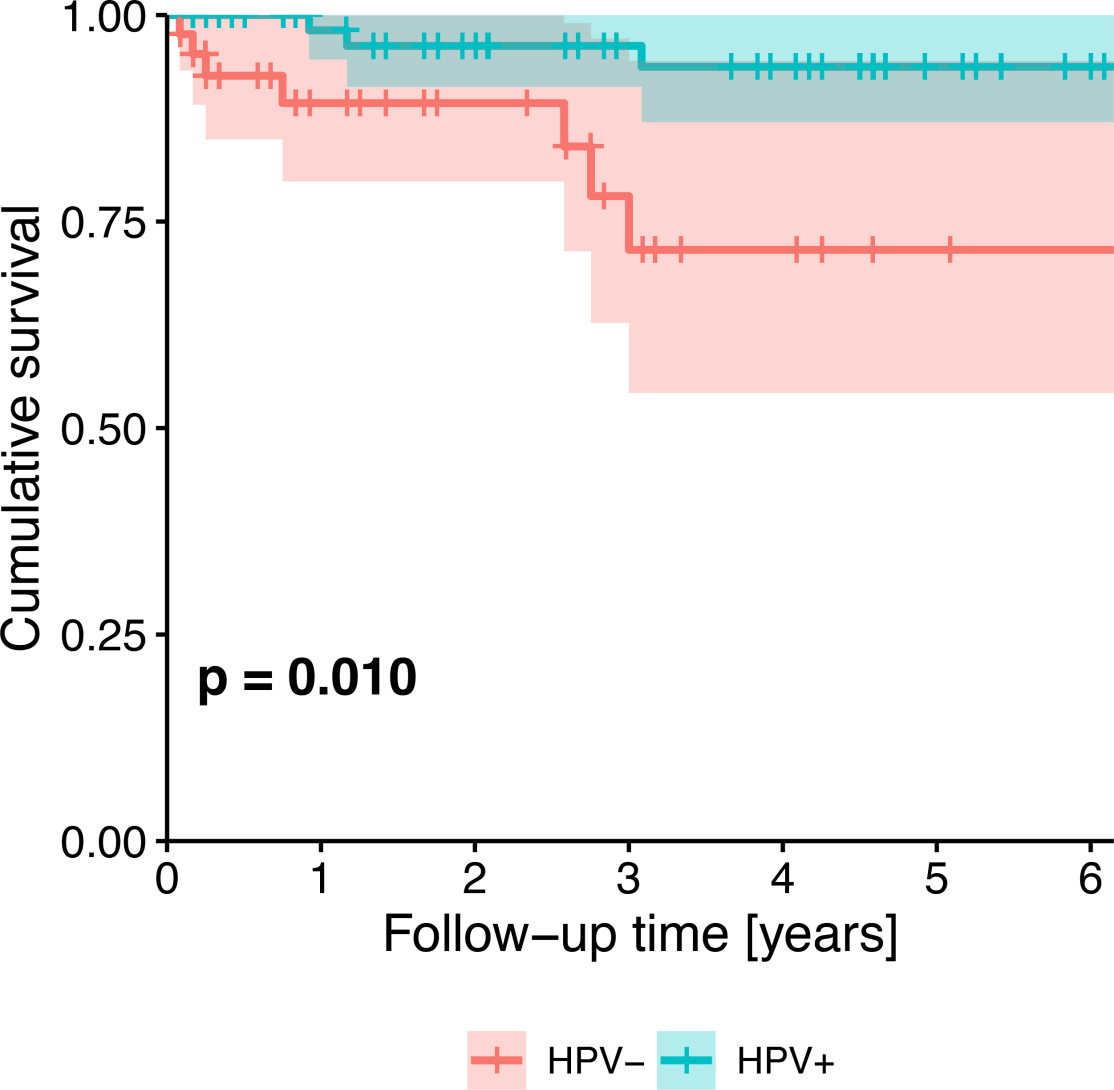
Kaplan-Meier survival plot showing the univariate effect of HPV status (HPV-negative patients vs. HPV-positive patients) on survival of all patients. P-value from likelihood ratio test against the null model.

**Figure 3:**
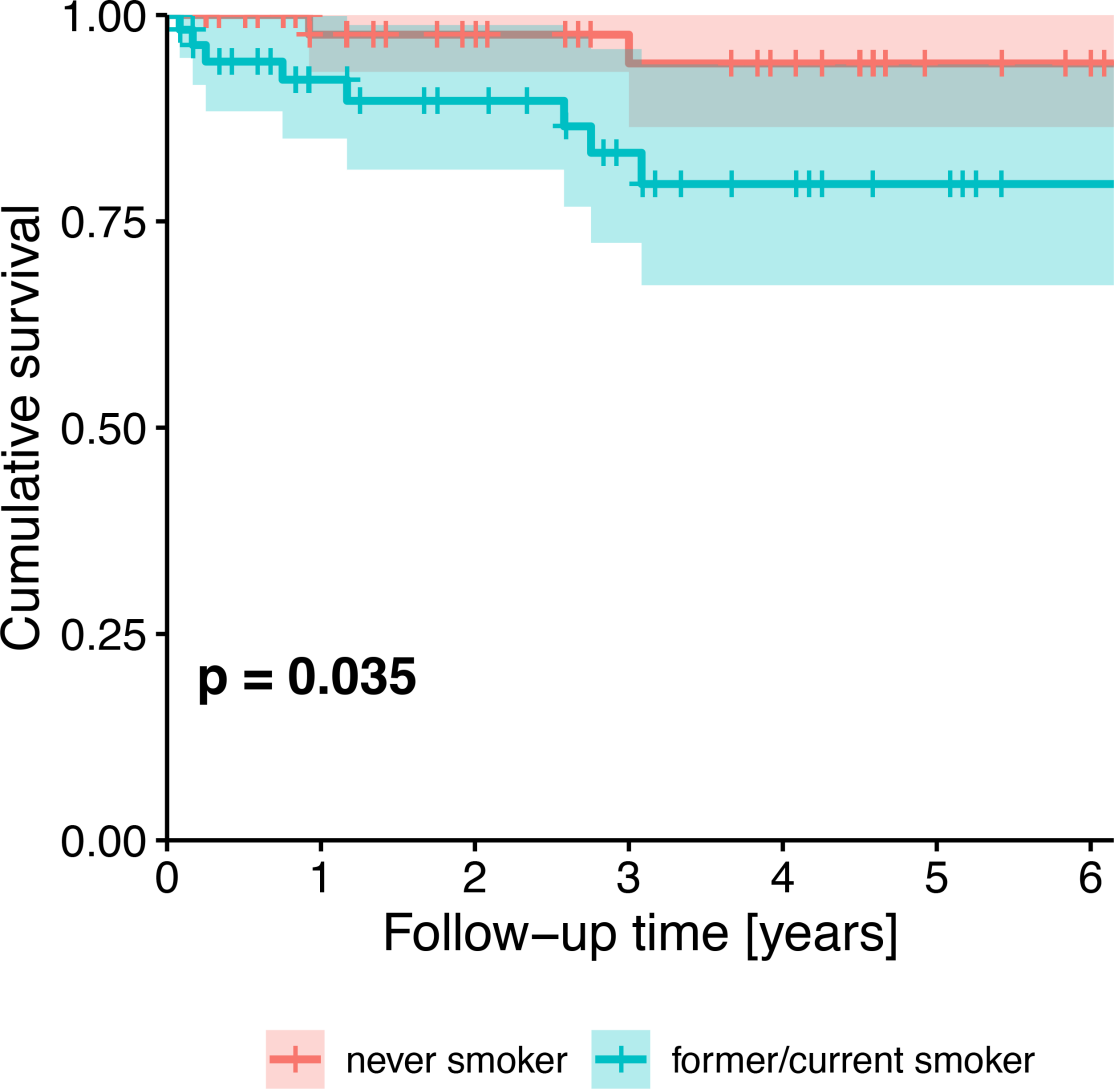
Kaplan-Meier survival plot showing the univariate effect of HPV status (never smoker vs. former/current smoker) on survival of all patients. P-value from likelihood ratio test against the null model.

Survival probability was significantly different between HPV-negative and HPV-positive patients (p=0.006). Five-year cumulative survival amounted to 71.6% (95%-CI: 54.2-94.4%) in HPV-negative patients and to 93.7% (95%-CI: 87.0-100.0%) in HPV-positive patients. Across all patients, survival trended towards being associated with HPV status (**Figure 2**), tobacco exposure (**Figure 3**), performance status, T stage, N stage, the number of missing and filled teeth as well as the number of root canal treated teeth with periodontitis apicalis and the number of teeth with periodontitis apicalis lacking a root canal treatment in dentulous patients (p≤0.077, **Table 2**). Within HPV-negative patients, only the performance status (p=0.015), T stage (p=0.055), and the number of teeth with periodontitis apicalis lacking a root canal treatment in dentulous patients (p=0.065) trended towards being associated with survival. Among HPV-positive patients, only the performance status (p=0.076), N stage (p=0.020) and the number of filled teeth (p=0.072) trended towards being associated with survival.

**Table 2:** Univariate effect (p-values) of patient-related risk factors for oropharyngeal squamous cell carcinoma, age, sex, and oral health parameters on survival.

		Total (n=119)	HPV-negative (n=50)	HPV-positive (n=69)
Age at diagnosis		0.670	0.996	0.894
**HPV status, HPV-positive vs. HPV-negative**		0.010 **	–	–
Sex, male vs. female		0.649	0.476	0.778
**Tobacco exposure, former/current smoker vs. never smoker**		0.035 **	0.530	0.256
Alcohol consumption, former/current vs. never		0.205	0.801	0.402
Primary treatment, surgery +/- adjuvant radiotherapy vs. radiotherapy/radiochemotherapy		0.188	0.300	0.649
Treatment intent, curative vs. palliative		0.402	0.745	0.783
**Performance status**		<0.001 **	0.015 **	0.076 *
**T stage**		0.023 **	0.055 *	0.810
**N stage**		0.048 **	0.330	0.020 **
M stage, M1 vs. M0		0.653	0.744	0.776
**DMFT**	D	0.248	0.958	0.156
	**M**	0.077 *	0.493	0.523
	**F**	0.003 **	0.148	0.072 *
	DMFT sum	0.456	0.839	0.901
**Number of teeth per patient**		0.077 *	0.493	0.523
Edentulous patients		0.212	0.687	1.000
Number of teeth in dentulous patients		0.223	0.575	0.523
**Root canal treated teeth in dentulous patients**	all	0.125	0.187	0.632
	without periodontitis apicalis	0.146	0.288	0.217
	**with periodontitis apicalis**	0.019 **	0.111	0.178
**Number of teeth with periodontitis apicalis but without root canal treatment**		0.032 **	0.065 *	0.305
Averaged radiographic bone loss in dentulous patients	mm	0.525	0.628	0.761
	%	0.456	0.508	0.783
	%/age	0.734	0.862	0.851

P-values from likelihood ratio test against the null model, * p<0.1, ** p<0.05.

For all patients, only tobacco exposure (p=0.004, HR=25.66), performance status (p=0.003, HR=0.87), and the number of teeth with periodontitis apicalis lacking a root canal treatment in dentulous patients (p=0.047, HR=1.65) remained significant in the multivariate analyses (**Table 3**). For HPV-positive patients, only performance status (p=0.037, HR=0.86) remained significant. For HPV-negative patients, none of the parameters remained significant in the multivariate analysis.

**Table 3:** Multivariate analyses including only variables with a p-value ≤0.1 within the univariate analyses.

		Total (n=119)	HPV-negative (n=50)	HPV-positive (n=69)
HPV status, HPV-positive vs. HPV-negative		0.692	–	–
**Tobacco exposure, former/current smoker vs. never smoker**		0.004 * HR: 25.66 (2.88-228.75)	–	–
**Performance status**		0.003 * HR: 0.87 (0.79-0.95)	0.073	0.037 * HR: 0.86 (0.75-0.99)
T stage		0.915	0.806	–
N stage		0.005	–	0.068
DMFT	M	0.342	–	–
	F	0.345	–	0.368
Number of teeth per patient		N/A	–	–
Root canal treated teeth in dentulous patients with periodontitis apicalis		0.999	–	–
**Number of teeth with periodontitis apicalis but without root canal treatment**		0.047 * HR: 1.65 (1.01-2.71)	0.606	–

The number of teeth per patient was not entered as an independent variable due to multicollinearity. For significant variables, hazard ratios (HRs) with their respective 95% confidence intervals are given. * p<0.05.

## 4 Discussion

This study has shown that OPSCC patients with positive HPV status have more present teeth, a lower DMFT and were less often edentulous than HPV-negative patients. Independent risk factors for survival of OPSCC patients were found to be former/current tobacco exposure, a low performance status, and teeth with periodontitis apicalis lacking a root canal treatment.

To date, limited information on the oral health of the specific group of OPSCC patients is available. In a recent study from Italy, the median DMFT of OPSCC patients amounted to 16, 82% of the patients were affected from periodontitis and only 2% were edentulous (1). Two studies from the UK reported a mean number of 19 to 22 present teeth, a DMFT of 15 to 17, and a moderate alveolar bone loss (4, 14).

With regard to the HPV status, Tezal et al. (15) compared HPV-positive and HPV-negative HNSCC (oral cavity, oropharynx, and larynx) patients. They found no differences between groups regarding caries, fillings, and edentulous state, but more bone loss and less missing teeth in HPV-positive tumors (15). In OPSCC patients, Patel et al. (14) found also less missing teeth in HPV-positive tumors, but also a lower DMFT and less horizontal bone loss compared to HPV-negative tumors. Farran et al. (24) reported less missing teeth and less patients with horizontal bone loss in HPV‑positive compared to HPV-negative OPSCC.

These observations are partly in line with the present study, also showing a lower DMFT and more present teeth in HPV-positive patients, but only slight and non-significant differences in periodontal bone loss between HPV-positive and HPV-negative patients. The relationship between smoking or alcohol abuse and periodontitis is well explored (25–27), and we found a higher percentage of smoking and alcohol consumption in HPV-negative patients, potentially accounting for a higher prevalence of periodontitis and – consequently – a higher number of missing teeth and a higher proportion of edentulous patients. However, missing teeth can act as surrogate parameter for periodontitis, but also for caries experience, and the reason for tooth loss was not available in the present study. Moreover, periodontitis and oral HPV-infection were also shown to be associated, and it is possible that periodontitis creates conditions that promote colonization and persistence of oral HPV infection (28). Taking into account that both groups present specific risk factors that might affect the development and progression of periodontitis, we found no significant differences with regard to periodontal bone loss.

Also, for both tobacco smoking and alcohol abuse a significant association with caries was shown (29, 30). While the causal relationship between caries and tobacco smoking and/or alcohol abuse is not fully elucidated, potential risk factors for the increased caries prevalence is the neglect of professional and oral care, especially in alcohol dependent people (31) as well as changes in the oral microbiome (32, 33) or salivary proteome (34). As a consequence of caries progression, not only the DMFT, but also the number of teeth with root canal treatment or in need for root canal treatment might increase. We found no differences regarding root canal treated teeth, but HPV-negative patients showed more teeth in need for root canal treatment. The findings of the present study indicate that HPV-positive patients have a better dental health, potentially due to a healthier lifestyle (less smoking, less alcohol consumption). Oral HPV-infection might also cause changes on the oral microbiome (35), but it is not known yet, whether this might affect caries development.

Previous studies regarding the effect of oral health parameters on survival of HNSCC patients found routine dental visits or good oral care behavior to be associated with a decreased mortality risk (5, 36). Only one study assessed the effect of oral health parameters on OPSCC patients and showed that the number of missing teeth and periodontal status predicted survival in univariate analyses. However, none of variables predicted survival in a full multivariate analysis (24). Correspondingly, no significant effect of oral health parameters on survival was found in the multivariate analysis of the present study, neither when all patients were taken into account, nor when HPV-negative and HPV-positive patients were analysed separately. The latter result is interesting as differences in dental health were detected between HPV-negative and HPV-positive patients. However, although the multivariate models were significant, it cannot be excluded that the overall analyses were affected by the limited follow-up periods. As a consequence, some of the well-established parameters predicting survival of OPSCC patients, e.g. HPV status (16–18) or T staging (37) were not significant in the multivariate analyses.

In this study, dental records of OPSCC patients that underwent a clinical and – if appropriate radiological – examination were included. Previous studies assessing oral health in OPSCC patients either used only radiographs (2, 4, 14) or self-reported proxy variables (e.g. tooth mobility, number of dental visits) (13), which can be considered as less valid compared to combined clinical and radiological examinations. Especially early caries stages are likely to be underestimated when only radiographs are used. Data on periodontal bone loss were obtained from radiographs, as previous studies found that both intra-oral and panoramic radiographs are suitable for determining alveolar bone loss or apical translucencies (19, 20). Data extraction and radiographic measurement were performed by one calibrated examiner, and the inter-rater and intra-rater reliability of the radiographic analysis was shown to be excellent, increasing the overall validity of our results.

However, as the present study was based on existing data, no clinical periodontal measurements (e.g. clinical attachment loss, inflammation status) and data regarding the quantification of alcohol and/or tobacco consumption were available. Also, further potential confounding factors, e.g. socioeconomic variables, were not available. Moreover, no data regarding the actually performed dental treatment (if required) were assessed. All included patients were referred for dental examination prior to cancer treatment. If required, dental treatment (i.e. extractions in most cases) was scheduled (if treatment was performed in the university) or recommended (if patients refused dental treatment in the university, but were treated by private dental practitioners). Therefore, we assume that only a limited number of patients received the necessary treatment in our department. As this study did not focus on the dental treatment, but on potential treatment needs, we did not assess potential dental treatment.

In this study, analysis was limited to OPSCC patients as these are most affected by different HPV status. Furthermore, only OPSCC patients prior to planned radio(chemo)therapy were included, as patients undergoing surgical therapy only are usually not referred for dental assessment. It is thus possible that the oral health within a more heterogenous group of OPSCC patients might be slightly different from the present cohort. In addition, comparisons between different anatomical sites (e.g. between oral cavity and pharynx) regarding HPV and survival are beyond the scope of the present manuscript and will be assessed in a separate publication.

## 5 Conclusion

HPV-positive OPSCC patients presented a better dental health compared to HPV-negative patients, indicating that tailored dental treatment is necessary for both subsets prior and during radio(chemo)therapy: HPV-positive patients present more teeth and are less often edentulous. Consequently, they might be more prone to dental complications during radiotherapy, specifically radiation caries (38). HPV-negative patients present a higher number of teeth with periodontitis apicalis, often making preradiation extractions necessary (39). A potentially severe consequence of preradiation extractions is osteoradionecrosis (40). To reduce long-term complications in both groups, prophylactic treatments including among others frequent topical fluoridation, regular dental visits, and oral care instruction are highly recommended (39).

## Data availability statement

The raw data supporting the conclusions of this article will be made available by the authors, without undue reservation.

## Ethics statement

The study was approved by the Ethics committee of the University Medical Center Göttingen. Written informed consent for participation was not required for this study in accordance with the national legislation and the institutional requirements.

## Author contributions

Conceptualization: SW and AW. Methodology: HS, DB and AW. Validation: PK., VH and AW. Formal analysis: PK. Investigation: KM. Data curation: PK. Writing—original draft preparation: PK and AW. Writing—review and editing: KM, VH, SW, HS and DB. Supervision: AW. All authors contributed to the article and approved the submitted version.
